# The Provision of a Punishment Book at Camberwell Infirmary

**Published:** 1913-11-22

**Authors:** 


### Camberwell Infirmary.

### THE PROVISION OF A PUNISHMENT BOOK.

The Camberwell Board of Guardians at their meeting last week again considered the petition presented to the Local Government Board for a public inquiry into the management of the infirmary. Mrs. Phillips, the chairman of the infirmary committee, presented the following report: The committee had under notice, by reference from the Board, letter from the Local Government Board, forwarding copy of a petition sent that Board. . . . Also drawing the attention of the Guardians to the regulations relating to punishments contained in the General Consolidated Order of December 8, 1847, in force in the parish, which prescribe amongst other things the conditions under which corporal punishment may be administered, and the keeping of a punishment book. The letter and petition having been read, it was ultimately resolved to recommend (*a*) that as the petition refers to matters which are the subject of an action between one of the signatories and the medical superintendent of the infirmary, no observations be made by the Board thereon at the present time, and that the Local Government Board be informed accordingly. The concluding paragraph of the letter as to the regulations relating to punishments was noted by the committee, and in connection therewith it was resolved to recommend (*b*) that a punishment book be provided for the infirmary for use in the future.

The paragraphs were discussed separately.

The Countess de Lormet said the action was a personal matter, and did not concern the Board.

The first recommendation of the committee was then carried, and the Board proceeded to discuss the provision of a punishment book.

The Countess de Lormet inquired if they had ever heard of a punishment book in a hospital or in any institution for the care of the sick. In the case of an inquiry at York, where a Special Commission sat to investigate certain questions of administration, it was proved that a nurse had smacked a child. For this she was dismissed. (Cries of "Shame.") The Consolidated Orders relating to punishments only dealt with workhouses and not infirmaries; and, even if it was assumed that they did refer to infirmaries, the Consolidated Orders did not apply to the Camberwell Infirmary. That infirmary was governed by a Special Order, which received the seal of the Local Government Board in 1900, two years after their infirmary was built, and was made expressly for their institution. This Order distinctly rescinded all previous Orders, and created it a distinct establishment. They could search the Order through, and they would find no sort or kind of punishment mentioned, or any clause giving power to any officer to punish any patient in any shape or form. Hence the proposal even to provide a punishment book was a violation of the Order governing Camberwell Infirmary. The recommendation that a punishment book should be provided was an admission that punishment had taken place. It proved that a *prima facie* case had been made out for a public inquiry.

Mr. S. Sayer hoped the Board would not agree to the proposal. A punishment book for sick people! He wondered what the ratepayers would say.

Mr. M. Boxall said he protested strongly against the whole tone of the Countess de Lormet's speech. She had tried to create prejudice against the administration of the infirmary. As a frequent visitor at the infirmary he had seen nothing of a repellent nature. He had never heard a single complaint against either the nurses or the medical staff.

Dr. R. Capes said the committee did not think that a punishment book was necessary, but thought perhaps it would be wiser to have one, so that if any punishment of any kind was inflicted they would have a record.

Mr. Roe and Mr. F. Grant opposed the provision of the punishment book.

Mr. S. H. Govier said he had never seen any child punished.

Mr. W. McCarthy said he hoped they would have a punishment book. In twelve months' time they would be able to see how they had been hoodwinked.

Mr. J. G. Spradbrow opposed all corporal punishment in any shape or form to sick children.

The Board, by twenty-three votes to five, carried the committee's recommendation that a punishment book should be provided.

